# Automation in the Construction of a 3D-Printed Concrete Wall with the Use of a Lintel Gripper

**DOI:** 10.3390/ma13081800

**Published:** 2020-04-11

**Authors:** Marcin Hoffmann, Szymon Skibicki, Paweł Pankratow, Adam Zieliński, Mirosław Pajor, Mateusz Techman

**Affiliations:** 1 Faculty of Mechanical Engineering and Mechatronics, West Pomeranian University of Technology, 70-310 Szczecin, Poland; ppankratow@gmail.com (P.P.); miroslaw.pajor@zut.edu.pl (M.P.); 2 Faculty of Civil Engineering and Architecture, West Pomeranian University of Technology, 70-310 Szczecin, Poland; adam.zielinski@zut.edu.pl (A.Z.); mateusz.techman@zut.edu.pl (M.T.)

**Keywords:** concrete 3D printing, digital construction, additive manufacturing, robotic fabrication

## Abstract

Developments in the automation of construction processes, observable in recent years, is focused on speeding up the construction of buildings and structures. Additive manufacturing using concrete mixes are among the most promising technologies in this respect. 3D concrete printing allows the building up of structure by extruding a mix layer by layer. However, the mix initially has low capacity to transfer loads, which can be particularly troublesome in cases of external components that need to be placed on top such as precast lintels or floor beams. This article describes the application of additive manufacturing technology in the fabrication of a building wall model, in which the door opening was finished with automatic lintel installation. The research adjusts the wall design and printing process, accounting for the rheological and mechanical properties of the fresh concrete, as well as design requirements of Eurocode. The article demonstrates that the process can be planned precisely and how the growth of stress in fresh concrete can be simulated, against the strength level developed. The conclusions drawn from this research will be of use in designing larger civil structures. Furthermore, the adverse effects of concrete shrinkage on structures is also presented, together with appropriate methods of control.

## 1. Introduction

The fact that the fourth industrial revolution has already come about, has been asserted in various sources [1]. It has been defined in terms of the integration of intelligent machines and systems and the implementation of changes in production processes, aimed at increasing productivity and improving changeover flexibility. The changes brought about by Industry 4.0 concern not only technology, but also new work methods and roles for the people involved. In particular, further developments will have an influence on areas such as artificial intelligence, robotics, the Internet of Things, additive manufacturing, nanotechnology and material engineering. The technologies mentioned above have functioned as initiation factors in the development of innovative solutions in the construction sector [2,3,4,5], which are related to the digital processing of new materials and technologies and the automated construction of buildings and structures [6].

**Figure 1:**
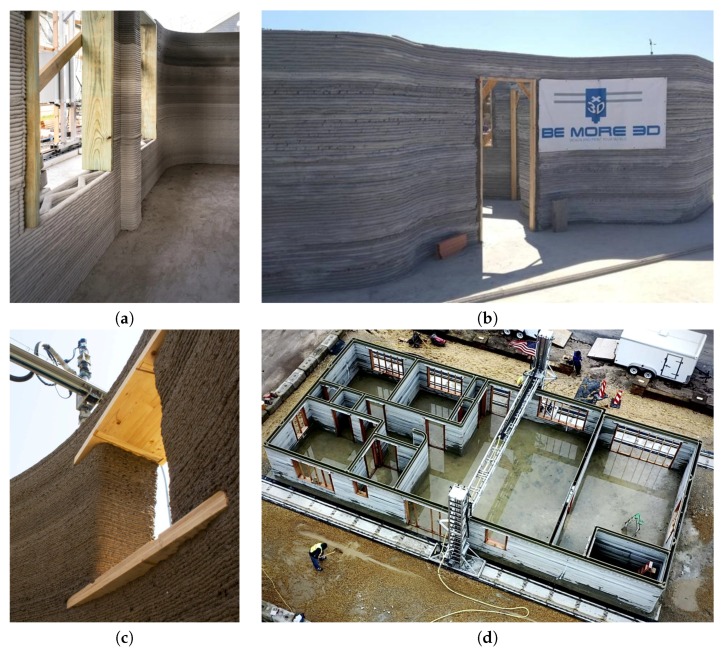
The use of timber shuttering for the production of building components, with the use of additive manufacturing technology: (**a**) ICON’s printed wall [20], (**b**) a construction by Be More 3D startup [21], (**c**) lintel in a window opening of a WASP 3D printed building [22], (**d**) SQ4D 3D Printed Home [23].

Researchers have paid particular attention to additive manufacturing technology, which has the potential of totally changing methods of construction using concrete and cementitious mortars. In this approach, the mix material is dispensed precisely at pre-determined locations, through a printing head nozzle. The printing head moves in 3D space over a programmed path, building up the designed structure layer by layer [2]. Various types of 3D printers are in use, including Cartesian robots [7,8,9], robotic manipulators [10,11,12] and Delta robots [13,14]. Several objects have already been produced with this technology, both for the purposes of demonstration as well as for practical uses [15,16,17,18,19]. In most cases, over the door and window opening timber shuttering has been used to support the deposited fresh concrete [20,21,22,23] (Figure 1). However, printing machines can also be used for the erection of precast elements, including lintels, floor beams and other structural components [24]. In these applications, the printer must be provided with the appropriate grippers, capable of handling such components.

In the additive manufacturing of structures, it is of primary importance to ensure a correlation between increase of the load caused by additional printed layers, and the growth of the strength of layers already placed during the process of curing. From this point of view, it is important to determine the appropriate extrusion speed, which makes it possible to obtain sufficient strength for each layer by the time the printing head returns to its home position, the layer needs to withstand the load imposed by layer deposited on top.

Challenge in 3D printing is to obtain a mix with desired rheological properties [3,25,26,27,28,29,30,31]. In the absence of standards for the assessment of the suitability of 3D printing mixtures, many research centers have developed their own procedures [3,25,26,30,31,32]. Mixtures are assessed in terms of extrudability, buildability, flowability, open time; i.e., parameters relevant for ensuring a proper printing process. Different approach has been taken by various research teams to evaluate the load bearing properties of fresh mix. Le et al. [26,32] evaluates the rheological properties of high-performance concrete using shear vane test which determines the shear strength. The mixes which shear strength ranges between 0.3 and 0.9 kPa are suitable in terms of extrudability and buildability. Other team [27] evaluates the suitability of the mix testing shape stability, which determines the deformation of the cylindrical specimen under 4.77 kPa stress in compression test. Another approach simulates a printed layer in the tests [33]. A cylindrical specimen with 60 mm diameter and 35 mm height was imposed with a load at certain intervals that reflects the consecutive layers printed in a real structure. The test allows determination of the time after which layers will collapse if imposed with a load. Certain, similar to ones used in soil mechanics test have also been applied, including uniaxial unconfined compression test [34] and direct shear test. A research [35] incorporated a cylindrical specimen with 60 mm diameter and 120 mm height for the purpose of the study. Numerical models for predicting variation in the stress and strain values of the individual layers of a structure as a whole, over time, are also being developed [32,34,35,36].

Considering that the formwork can constitute to 35–54% of total costs of raising concrete structure [30,37,38,39]. The application of the additive manufacturing brings measurable profits. Not only does it allow production of structures without the formwork, but it also reduces total production time, costs and labor. The technology also increases the safety of workers on the construction site, produces less waste and uses raw materials with low embodied energy [30,39].

The purpose of this article is to present the process of the additive manufacturing of a scale down model of a wall with a door opening, including the installation of a lintel by means of a specially designed gripper. The research adjusts the wall design and printing process, accounting for the rheological and mechanical properties of the fresh concrete, simultaneously taking into account the process of lintel installation. The article will demonstrate that the process can be designed with high accuracy, as confirmed by simulation.

## 2. Materials, Methods and Experiment Program

### 2.1. 3D Printer

**Figure 2:**
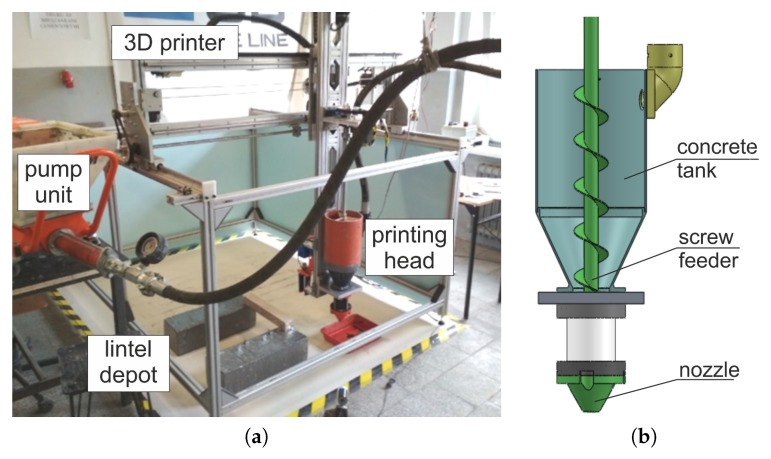
3D printing set-up: (**a**) Cartesian robot and pump unit, (**b**) printing head.

The wall was constructed at a specially designed site, consisting of a 3D robot connected to a pumping module (Figure 2a). The mix was extruded by a screw feeder (Figure 2b), with D = 20 mm nozzle outlet diameter. The printer and printing head motions were controlled by a G-code. The mix was prepared in a laboratory mixer and transferred to the pump unit, from where it was delivered to the printing head hopper through a hose.

### 2.2. Gripper Design

**Figure 3:**
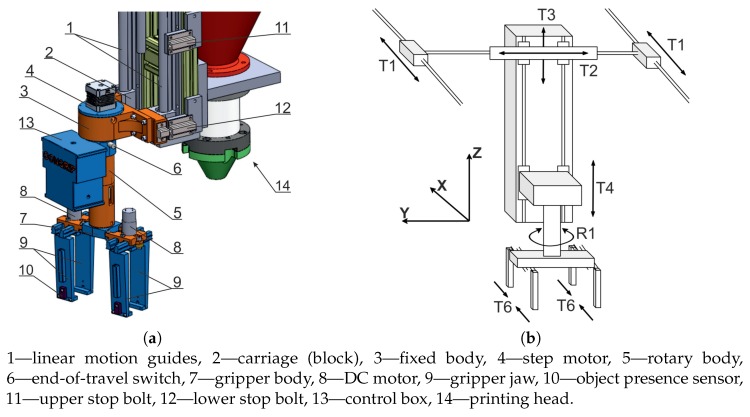
Lintel gripper construction: (**a**) printer and gripper kinematic diagram, (**b**) mounting of the gripper onto Z axis of the 3D printer.

As part of this experiment, a special gripper was built to transfer the lintel from the lintel depot and install it on the wall in determined position. These operations took place when the printing robot was running, with the printing process recommencing as soon as they were completed. The gripper construction was integrated with the 3D printer structure, with the gripper being mounted at the end of the Z column of the printer, which also supported the printing head (Figure 3a). The gripper structure did not interfere with the head movements, during the building of the concrete structure. During the operation of the printer, the gripper adopted the degrees of freedom of the Cartesian robot: T1, T2, T3 linear motions along the X, Y, Z axes, respectively (Figure 3b). The T4 was an auxiliary motion used to move the device outside the printer. The vertical motion of the gripper was affected by the T3 printer motion. Rotational motion R1, about the Z axis, rotated the assembly, comprised of two grippers. The motions of the T6 gripper jaws were synchronized.

The design of the gripper is presented in Figure 3a. The carriages (2), on which the fixed gripper body is mounted, travel on the linear motion guides (1). The step motor (4) rotates the body (5), the position of which is determined by a limit switch (6). The gripper body (7) accommodates DC motors (8), which operate the gripper jaws (9). Presence sensor (10) are attached to the gripper jaws. The jaw mechanisms can withstand a force required to carry a lintel of up to 16 kg in weight. The retracted and extended positions of the gripper, are determined by the positions of the upper (11) and lower stop bolts (12) (T4 motion, as shown in in Figure 3b). The control system (13) is based on an Arduino Leonardo microcontroller. It operates all gripper drives, receives signals from IR interrupt sensor and from the end-of-travel limit switch. The gripper motions are controlled by the G-code instructions, which trigger the successive functions required to build the designed concrete wall.

### 2.3. Procedure for Building a Concrete Wall Incorporating a Lintel

**Figure 4:**
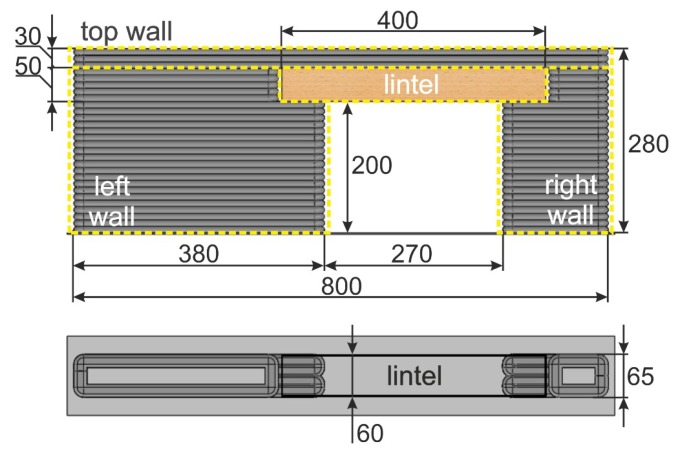
The dimensions of the printed wall.

A model of a printed wall, with dimensions, has been presented in Figure 4. The design assumed that the wall would be built up by the deposition of $h_{l}$ = 10 mm × $w_{l}$ = 25 mm layers, by a printing head travelling at a speed of F = 3500 mm/min. The construction was divided into four sections: left wall, right wall, lintel and top wall.

**Figure 5:**
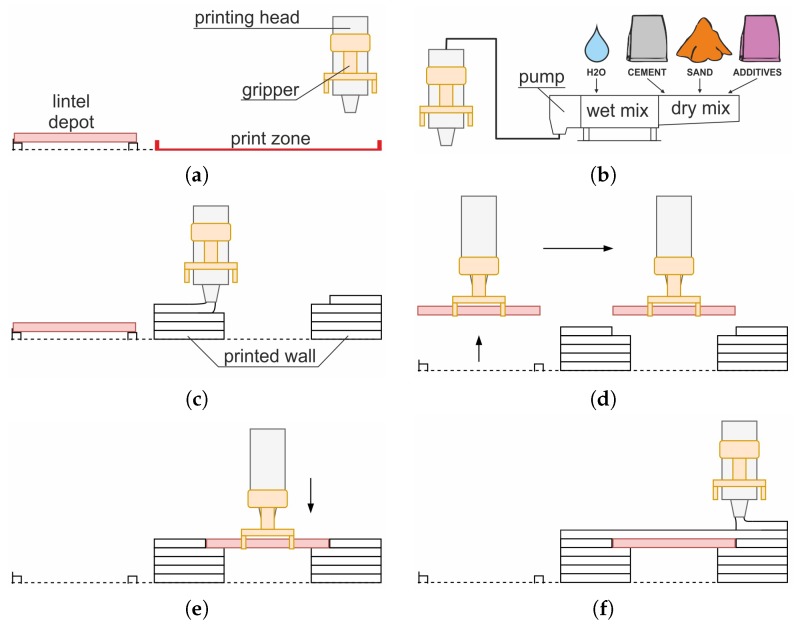
The steps for printing a wall with a lintel: (**a**) Step 0—preparatory activities, (**b**) Step 1—concrete mix preparation, (**c**) Step 2—printing of the wall, (**d**) Step 3—lintel picked up from the depot, (**e**) Step 3—installation of lintel, (**f**) Step 4—continued printing.

The construction process was divided into five steps, with the preliminary step being comprised of all the preparatory activities, followed by four production steps:

- Step 0: set up all the process elements, i.e., lintel depot, 3D printer, printing head, lintel gripper and process control code (Figure 5a);
- Step 1: prepare 25 L of concrete mix, estimated time: 15-20 min (Figure 5b);
- Step 2: print left and right wall sections (Figure 5c), estimated time (G-code printing process simulation): 12 min;
- Step 3: pick up the lintel from the depot (Figure 5d) and place it over the door opening (Figure 5e), estimated time: 4–7 min;
- Step 4: deposit new layers of concrete on the lintel, thus building up the top wall (Figure 5f), time estimated on the basis of G-code simulation: 2 min.

The operational time of particular stages has been established based on the G-codes for the printer and grabber.

### 2.4. Concrete Mix

The concrete mix used in the experiments was designed on the basis of article [9], with a 0.23 water-cement ratio and a density of 2168 kg/m${}^{3}$ (CoV = 0.24%, n = 3). The binder was composed of: (1) CEM I 52.5R Portland cement −70%; (2) fly ash −20%; (3) silica fume −10% of the total binder amount. A fine aggregate (0–2 mm grading) was used. A 28-day strength of 113.7 MPa (CoV = 1.13%, n = 4), was obtained for the mix. Previous qualitative tests have shown that the open time of the mix ranges from 10 to 70 min [9]. During the mix preparation and printing operations, the ambient temperature was 20 ${}^{\circ }$C ± 2 ${}^{\circ }$C, while the relative air humidity was 60% ± 5%.

## 3. Design of the Printed Wall Structure

The lintel was designed as a *b* = 60 mm wide by *h* = 50 mm high and *L* = 400 mm long LVL beam with crossband veneers; class LVL 32 C, as per EN 14374:2004 [40], with 11% ± 1% moisture content. The remaining parameters of the designed lintel beam are presented in Table 1, below.

**Figure 6:**
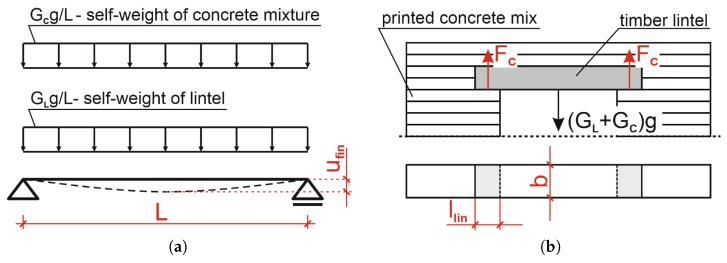
(**a**) Lintel loading diagram used for calculating the amount of deflection, (**b**) lintel support, load and geometry.

The stress distribution under the lintel was determined based on the calculated deformations. For the purposes of these calculations, the lintel span was assumed to be equal to its length. The loading diagram, including the self-weight of the lintel and the weight of the fresh concrete deposited on the top of it, has been presented in Figure 6a. The beam weight was $G_{L}$ = 0.612 kg, while the weight of the fresh concrete placed on top of it was $G_{C}$ = 2.592 kg. The lintel deflection $u_{fin}$ was calculated assuming a span to height ratio of *L/h* < 20 (National Annex [41]). For the loading and lintel geometry adopted, the deflection of the lintel, calculated with Equation (1) [41] was 0.005 mm. Such a value was obtained due to the high stiffness of the lintel beam. As such, the stress distribution over the lintel was assumed to be uniform (i.e., without concentrations). The lintel loading diagram and assumed dimensions, has been presented in Figure 6b.

(1) $$u_{fin}=\frac{5}{384}\frac{\left(G_{L}+G_{C}\right)\cdot g\cdot L^{3}}{\left(E_{0,mean}\cdot I_{y}\right)}\left(1+19.2{\left(\frac{h}{L}\right)}^{2}\right)$$

where $u_{fin}$—lintel beam deflection, $G_{L}$—beam weight, $G_{C}$—weight of concrete deposited over the beam, $E_{0,mean}$—beam modulus of elasticity, *L*—beam span, *h*—lintel beam height, $I_{y}$ = moment of inertia. The level of stress at the lintel support point $\sigma_{c}$ was calculated using the following Equation (2):

(2) $$\sigma_{c}=\frac{F_{c}}{A_{c}}=\frac{(G_{L}+G_{C})\cdot g}{\left(2\cdot b\cdot l_{lin}\right)}$$

where $\sigma_{c}$—stress at lintel support point, $F_{c}$—reaction at the lintel support, *g* = 9.81 m/s${}^{2}$ is the gravitational acceleration, $A_{c}$—lintel end bearing area, *b*—lintel width, $l_{lin}$—end bearing length on one side.

The calculated stress needs to be lower than the maximum stress $\sigma_{max}$ that can be sustained by the already deposited material. Moreover, the values of $\sigma_{c}$ and $\sigma_{max}$ depend on the age of the fresh concrete, i.e., time *t*. This time is measured from the adding of water until the achievement of a dry mix. The strength of the lintel bearing area was calculated using Formulas (3) and (4).

(3) $$\sigma_{c}\left(t\right)\leq \sigma_{max}\left(t\right)$$

(4) $$\frac{F_{c}\left(t\right)}{b\cdot l_{lin}}\leq \sigma_{max}\left(t\right)$$

Given the lintel end bearing width *b*, Equation (4) can be rearranged to calculate the end bearing length $l_{lin}$:

(5) $$l_{lin}\geq \frac{F_{c}\left(t\right)}{b\cdot \sigma_{max}\left(t\right)}$$

**Figure 7:**
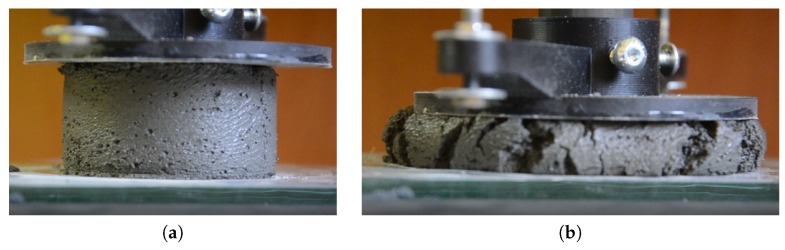
(**a**) Sample during the test and (**b**) after the test.

The next step involves determining the allowable stress level in the deposited fresh concrete $\sigma_{max}$ as a function of time *t*. The mechanical properties of the concrete mix were determined using a test set-up, simulating the printing process [33]. A sample of fresh concrete, 35 mm high by 60 mm in diameter, was placed between two parallel surfaces and a load of 150 g was applied incrementally on the top surface. The load plate displacement was measured with LVDT inductive sensors. These measurements were used to determine any sample deformations. Figure 7a represents a view of the sample during load application, with Figure 7b representing the sample after failure.

**Figure 8:**
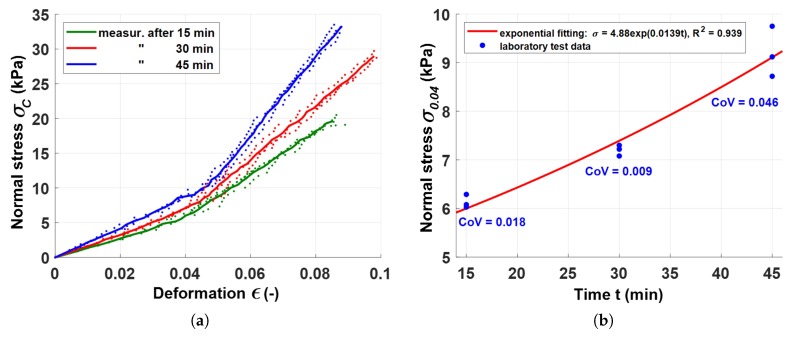
Experimentally determined mixture behavior (**a**) relationship between stress and deformation, (**b**) level of stress at a strain of 0.04, depending on the age of fresh concrete.

The test was programmed to increase the load by amounts simulating increases in load from the application of successive layers of concrete, as they are deposited in reality at 10 s intervals. The test was carried out in three variants: (1) 15 min after adding water, (2) 30 min after adding water, (3) 45 min after adding water. The slump value of the tested concrete mix was in the range of 150–165 mm. Separate samples were prepared for each time variant of the test. Figure 8a presents the relationship between stress $\sigma_{c}$ and strain ϵ in compression test for the mix, for assumed time variants. The solid line represents the average value of the three measurements, while the dotted lines represent the results of each of the three tests, respectively. In each case, failure of the samples occurred at the limit strains of 0.08 to 0.10. The failure stress values corresponding to the ultimate strains, depended on the case analyzed and, as expected, increased with the amount of time that lapsed from the moment of adding water to dry components. Strains in the region of the observed limit strains, if allowed during the printing of real structures, could cause excessive settlement of the construction under the weight of the deposited fresh concrete and the installed precast elements. Moreover, as can be seen in the graphs in Figure 8, at strains of around 0.04, the stiffness of the fresh concrete increased, as indicated by a change in the curve inclination angle. It was therefore decided to limit the level of stress to a value defined by a strain level of 0.04 (6).

(6) $$\sigma_{max}=\sigma_{0.04}$$

The adopted maximum allowable strain level of 0.04 is acceptable, in the authors’ opinion, and should not have a significant bearing on the strain conditions of the structure under consideration. The relationship between a stress value of 0.04 ($\sigma_{0.04}$) and the age of fresh concrete at the start of testing (for *t* = 15, 30 and 45 min) (Figure 8b), was also determined. A power function was used to describe the $\sigma_{0.04}$(t) relationship, as proposed in [33]. The graphs below show the Coefficient of Variance (CoV) and the coefficient of determination (R${}^{2}$) of the function analyzed.

Given the relationships (5) and (6), it is possible to calculate the minimum bearing length of the lintel $l_{lin}$. This value will be defined by the whole printing process and depend heavily on loading changes over the course of the process. Please note that the reaction force from lintel $F_{c}$ changes linearly, as the wall building process proceeds (lintel installation followed by the depositing of successive layers). An exponential function is, in turn, used to describe the changes in stress $\sigma_{0.04}$. The relationship defining the minimum value of $l_{lin}$ is expressed by the following equation:

(7) $$l_{lin}\geq \frac{F_{c}\left(t\right)}{b\cdot \sigma_{0.04}\left(t\right)}$$

To increase the chances of successful printing, the minimum end bearing length $l_{lin}$ was designed using load combinations and safety factors, in compliance with the safety standards indicated in [42,43]. A combination of actions was adopted, using Equation (6.10), for set B (design values of actions STR/GEO), as per EN 1990:2002 [42].

The most unfavorable combination was assumed, including both permanent and live loads. The lintel weight $G_{L}$ was taken as the permanent load, with a safety factor of $\gamma_{Gj,\sup}$ = 1.35 [43] applied to it. The weight of the layers of concrete deposited on the lintel, $G_{C}$ increased over time and can be assumed as variable action to the process. In this case, a safety factor of $\gamma_{Q,1}$ = 1.5 [43] for this action can be used. Based on these structural assumptions, the minimum end bearing length $l_{lin}$ of the lintel was calculated with Equation (8):

(8) $$l_{lin}\geq \frac{(1.35G_{L}+1.5G_{C}\left(t\right))\cdot g}{b\cdot \frac{\sigma_{0.04}\left(t\right)}{1.35}}$$

**Figure 9:**
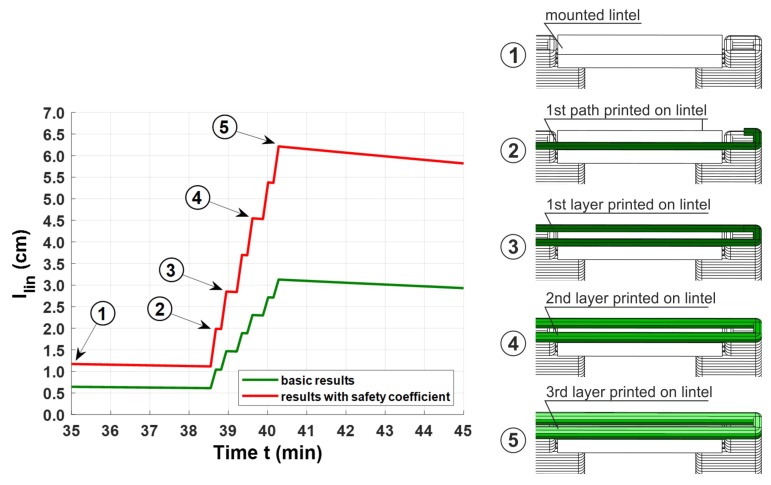
Minimum end bearing length as a function of time, modified by safety factors included in Equation (8) (red line) and the results ignoring the safety factors (green line) calculated with Equation (7).

Figure 9 presents the relationship between the minimum end bearing length and the time obtained from Equation (8) (red line). It was assumed that the lintel would be installed in the 35th minute, after water was added to the mix. As soon as the gripper had completed its job, the printing head was prepared, so as to commence depositing the first layer of the top wall section over the lintel, in the 38.5th minute. When the first layer is deposited (point 3 in Figure 9), it induces a stress value $l_{lin}$ of at least ca. 2.8 cm in the fresh concrete. According to the printing process simulation, covering all the three layers, the minimum end bearing length should not be smaller than 6.3 cm; thus a value of 6.5 cm was finally adopted for $l_{lin}$. The minimum end bearing lengths, calculated with Equation (7) but without the application of safety/partial factors according to [42], are presented in Figure 9, for the sake of comparison (green line).

## 4. Experimental Results and Discussion

**Figure 10:**
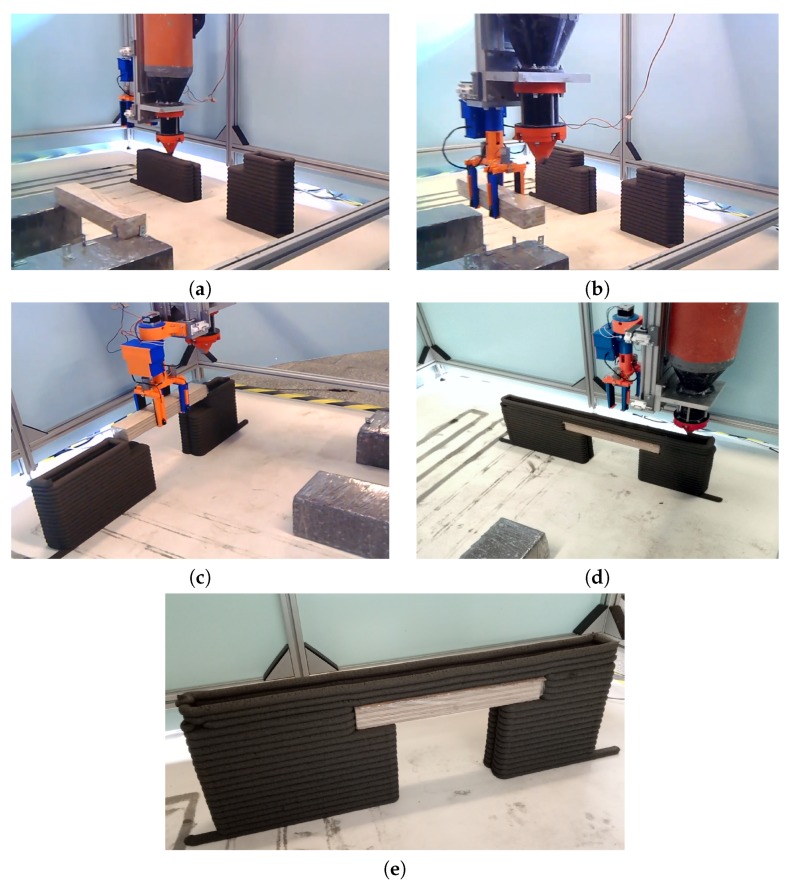
Steps of the wall printing process: (**a**) printing of the wall sections—step 2, (**b**) manipulator picking up the lintel—step 3, (**c**) installation of the lintel in the wall opening—step 3, (**d**) printing of the top wall section—step 4, (**e**) completed 3D-printed wall.

The designed wall was built with a semi-automatic process, according to the above-described procedure. Figure 10 presents the respective steps of the wall construction process. Step 0 included preparation and installation of all the devices required for the job, setting up the lintel depot and defining the lintel location in the printer reference system, determining the printing zone location, testing of the G-code during test printing and lintel installation (Figure 10a). The wall construction process started with the preparation of a fresh concrete mixture (Step 1), which took 15 min. Step 2, which took 14 min, was comprised of printing the left and right wall sections, including the lintel supports. Step 3, which was completed in 10 min, involved setting up the gripper and initiating it to move to the lintel depot to pick up the lintel (Figure 10b), to install the lintel in the wall (Figure 10c) and then to move back and retract. This step took longer than initially planned, due to the necessity of adjusting the gripper position during the lintel installation operation. Finally, the lintel came to rest on the supports in the 38th minute, after water was added to the mix. Step 4, that of printing the top wall section, was completed in 2 min (Figure 10d). Figure 10e presents an overview of the completed wall, including the lintel.

**Figure 11:**
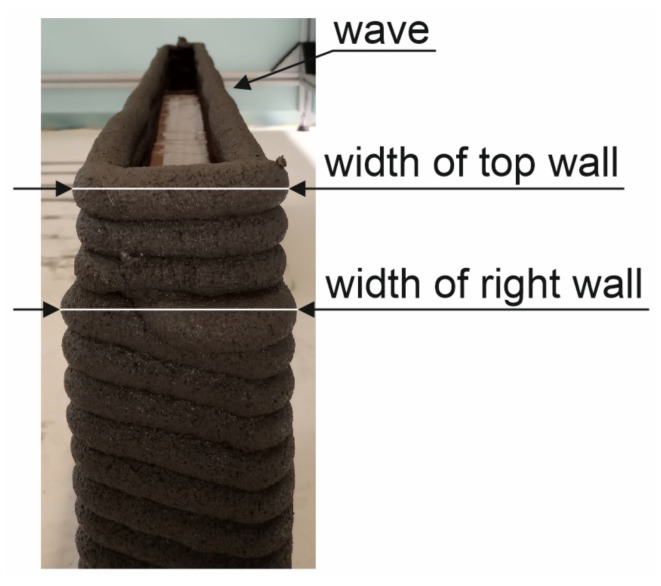
Construction of the 3D printed wall, immediately after depositing a layer of the top wall section.

Several problems were encountered during the process. Figure 11 presents a side view of the completed 3D-printed wall, highlighting the uppermost layers. During lintel installation, the mix remained unstirred in the hopper for 10 min. This caused greater than expected loss of moisture as the top of the hopper was opened. As a result, the viscosity of the mix increased drastically affecting the extrudability. The upper layers were therefore comparatively thinner than the bottom ones. Figure 11 shows the changes in shape of the uppermost layer; attributable to a high mix viscosity. Increase of the viscosity caused reduces slump resulting in decreased contact area between the deposited layers. This in turn caused overhangs to appear. The rotary motion of the screw feeder can also be blamed for the reduced extrudability and discontinuations in the deposited layer. The problem can be solved by applying a monitoring system for rheological properties shown e.g., in [44,45]. The automatic system determines the properties of fresh mix in real time and adjusts the flow rate and printing speed within the feedback system. The solution results in a good quality of the printed path without holes and discontinuations.

**Figure 12:**
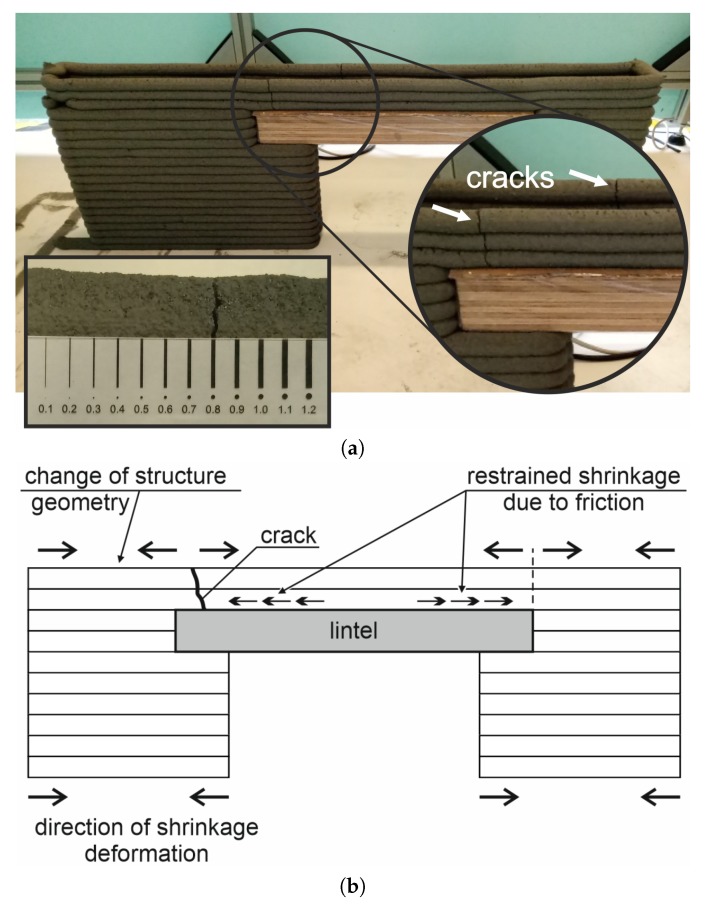
Steps of the wall printing process: (**a**) printing of the wall sections—step 2, (**b**) manipulator picking up the lintel—step 3, (**c**) installation of the lintel in the wall opening—step 3, (**d**) printing of the top wall section—step 4, (**e**) completed 3D-printed wall.

The most serious problem was the cracking observed in the uppermost layers of the printed structure, one day after completion of the printed structure (Figure 12a). Initial small cracks appeared in the top wall section over the lintel which later developed into structural cracks. The cracks occurred close to the upper corners of the opening and were caused by shrinkage restraint. Two different shrinkage mechanisms were involved in this case: plastic shrinkage and autogenous shrinkage [46]. Plastic shrinkage being drying kind of shrinkage of a freshly placed mixture, results from the dynamic tendency towards equilibrium, between the relative humidity of the ambient environment and the moisture content of the material. Autogenous shrinkage, in turn, reduces the volume of the material as a result of chemical reactions in the binder hydration process. Both these shrinkage types occurred very intensively in the initial hours after extrusion. The fresh concrete started to dry right after placement, when it had not, as yet, developed any mechanical resistance properties. The dynamic action of autogenous shrinkage related to the commencement of the setting process, additionally increased drying shrinkage, adding to the drying and autogenous shrinkage deformations. Furthermore, overall shrinkage was restrained, due to the following three factors (Figure 12b): (1) the presence of a rigid lintel beam located over the opening, which acted as a restraint to the shrinking material, (2) the forces of friction between the concrete mix and the lintel beam surface also acted as shrinkage material, (3) the left- and right-hand wall sections were printed in one operation and, as such, their shrinkage involved uniform deformation towards the cross-section core. The dead weights of these wall sections acted as a constraint to the shrinkage of the overlying layers.

Shrinkage restraint resulted in the development of internal stresses in the fresh concrete. When the dynamically increasing shrinkage stresses exceeded the early-age tensile strength, cracking occurred [47]. The direction of local deformations and crack locations are presented in Figure 12b. As shrinkage progressed continuously, the element lost structural integrity at the crack locations, with the cracks widening to about 0.8 mm on the day after placement (Figure 12a). The width of shrinkage cracks resulted from low relative humidity of RH = 60%, on the three sides of the deposited fresh concrete with a high content of mineral binder (820 kg/m${}^{3}$).

The reduction in discontinuations and cracking caused by shrinkage is one of the major challenges for this technology [31]. Shrinkage can be minimized by means of internal or external curing. Internal curing includes (without limitation): the use of shrinkage reducing admixtures, water-saturated lightweight aggregates and adsorptive fibers [46,47,48,49]. External curing techniques include the application of water, or a membrane forming curing compound, on the printed surface. Any of the above-mentioned measures can be applied to mitigate cracking of concrete and can effectively protect printed layers from tight shrinkage cracks, which subsequently develop into structural cracks. However, these curing related modifications require additional testing.

## 5. Conclusions

The article has presented the whole construction process, divided into preparation and construction steps. An important part of this study was to present a 3D printed wall design procedure, which took into account variance in the mechanical properties of the cement-bound composite material, over time. Attention is drawn to the following design aspects, which are considered to be part of the design process:

- Both permanent and variable actions should be taken into account when calculating the stresses induced at the lintel, or at other structural element locations. Permanent actions are due to the weights of precast elements, while variable actions come from the layers of concrete deposited in the printing process.
- It is necessary to determine the maximum allowable strain of the cement-bound material, resulting from the loads applied. In this study, an ultimate strain at the level of 0.04 was adopted. This value ensured that settlement of the construction under the imposed load, would not exceed an acceptable, benchmark level.
- The adopted maximum strain criterion can be used to determine the maximum permitted stress level, in the concrete mix used for the process. Note that the maximum permitted stress levels increase over time. In the calculations above, this was demonstrated by a decreasing lintel end bearing length value $l_{lin}$, due to an increase in the strength of the fresh concrete. This effect was evident in the size simulation.
- A highly rigid lintel beam will allow the assumption of uniform distribution of stress over the end bearing area.
- The shrinkage of the concrete should be considered for 3D printed structures as it causes loss of structural integrity or cracking during curing.
- During the process of printing, particular attention should be paid to concrete mix consistency. Increased viscosity will affect extrusion of the mix and the quality of the completed 3D-printed structure. Measures for maintaining mix viscosity at the desired level include, continuous mixing, protection from excessive loss of moisture, using smaller batches of material and continuous production.

The findings of this research confirm the possibility of automating the wall construction process in additive manufacturing technology, including the installation of a lintel with a special gripper.

- Increasing the number of automatic operations in the process of additive manufacturing is one of the solutions for reduction of total costs of the investment [31].
- It was proven that the printer can be used for placing of precast elements during the whole process of printing.
- The installation time of the lintel should be as short as possible to limit the influence of the delay on the rheological properties of mix in the pumping system.
- The pumping installation can be upgraded with monitoring system for determination of real time rheological properties. The data can be used to e.g., steer the pumping and printing speed to produce a path of assumed dimensions [44,45].

In summary, a lack of design and testing methods developed specifically for concrete and mortar printed structures is an important issue. The authors proposed to incorporate the current Standards and basics of structural mechanics for 3D printing. Used Eurocode are normally applied for structural design and calculation of timber and composite structures [40,41,42,43]. The rheological properties of fresh concrete are of higher significance, than in the case of conventional concrete construction, due to the specific process characteristics involved. Generally, the mixes used for 3D printing feature a higher binder content and lower water-binder ratio, which result in high shrinkage, including autogenous shrinkage, of fresh concrete. There are currently several experimental projects being carried out at different research centers, investigating the desired mechanical and rheological properties of concrete mixes and their behavior during the printing process. Further research concerning mix properties and 3D printing strategies will result in the development of design and construction procedures, which will ensure the required level of printed construction structural safety.

## Figures and Tables

**Table 1:** LVL beam parameters.

Parameter	Unit	Value
γ—specific weight	kN/m${}^{3}$	5.1
$E_{0,mean}$—modulus of elasticity	kN/cm${}^{2}$	1000
$I_{y}$—moment of inertia	cm${}^{4}$	62.5
